# Humoral immune response of pigs, *Sus scrofa domesticus*, upon repeated exposure to blood-feeding by *Ornithodoros turicata* Duges (Ixodida: Argasidae)

**DOI:** 10.1186/s13071-020-3931-8

**Published:** 2020-02-12

**Authors:** Hee J. Kim, Aparna Krishnavajhala, Brittany A. Armstrong, Adalberto A. Pérez de León, Serhii Filatov, Pete D. Teel, Job E. Lopez

**Affiliations:** 1 Department of Entomology, Texas A&M AgriLife Research, College Station, TX USA; 2 Department of Pediatrics, National School of Tropical Medicine, Baylor College of Medicine, Houston, TX USA; 3 Department of Molecular Virology and Microbiology, Baylor College of Medicine, Houston, TX USA; 4 Knipling-Bushland U.S. Livestock Insects Research Laboratory and Veterinary Pest Genomics Center, United States Department of Agriculture, Agricultural Research Service, Kerrville, TX USA; 5 Laboratory of Virology, National Scientific Center, “Institute of Experimental and Clinical Veterinary Medicine”, Kharkiv, Ukraine

**Keywords:** *Ornithodoros*, Western blotting, Enzyme-linked immunosorbent assay, Vector-host interactions

## Abstract

**Background:**

*Ornithodoros turicata* is an important vector of both human and veterinary pathogens. One primary concern is the global spread of African swine fever virus and the risk of its re-emergence in the Americas through potential transmission by *O*. *turicata* to domestic pigs and feral swine. Moreover, in Texas, African warthogs were introduced into the state for hunting purposes and evidence exists that they are reproducing and have spread to three counties in the state. Consequently, it is imperative to develop strategies to evaluate exposure of feral pigs and African warthogs to *O. turicata.*

**Results:**

We report the development of an animal model to evaluate serological responses of pigs to *O. turicata* salivary proteins after three exposures to tick feeding. Serological responses were assessed for ~ 120 days by enzyme-linked immunosorbent assay and immunoblotting using salivary gland extracts from *O. turicata.*

**Conclusions:**

Our findings indicate that domestic pigs seroconverted to *O. turicata* salivary antigens that is foundational toward the development of a diagnostic assay to improve soft tick surveillance efforts.

## Background

**Fig. 1:**
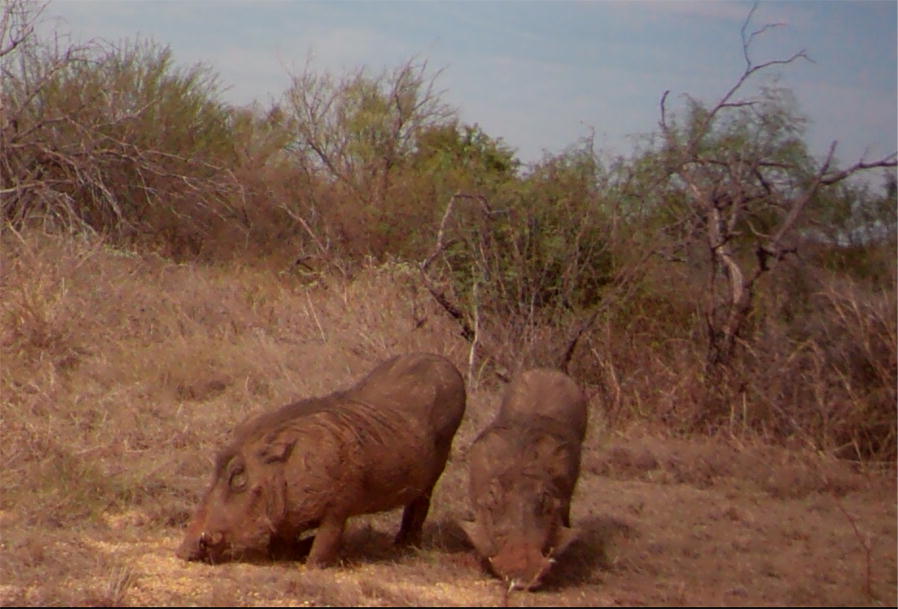
Photographic images of two African warthogs taken using a Moultrie game camera. Images were taken in La Salle County, Texas, USA

*Ornithodoros turicata* Duges is a nidicolous, cavity dwelling argasid tick found in the southwestern USA, Mexico, and Florida [1–3]. This soft tick species is recognized as a vector of human relapsing fever, caused by the spirochete *Borrelia turicatae* [4, 5]. Additionally, laboratory studies documented the potential role of *O*. *turicata* as a vector of other pathogens including African swine fever virus (ASFV) [6]. ASFV is a highly contagious pathogen of swine and recognized as a select agent by the USDA. First discovered in 1920 in Kenya, ASFV was found to have a sylvatic cycle that principally involves two species of African warthogs, *Phacochoerus aethiopicus* Pallas and *Phacochoerus africanus* Gmelin (both Artiodactyla: Suidae) [7]. These species also serve as hosts for *O. moubata*, the argasid vector. ASFV moved into Spain, in the 1960s and remained enzootic in the region sustained in part by the vector *O. erraticus*, until it was eradicated [8]. Recent expansions of ASF through the Caucasus region, into Europe, and most recently to Asia have increased concerns of a global threat to the swine industry [9]. Importantly, in the USA, African warthogs were imported into Texas as wild game. Recently, the animals have escaped and been spotted in three counties within the state (Fig. 1) and are reproducing. This signifies an ecological setting for the emergence of ASFV in the USA.

The sylvatic cycle of ASFV in Africa produces little to no clinical manifestations among infected warthogs, or the limited feral swine that recover from infection [9, 10]. However, both ASFV-infected vectors and the sylvatic hosts can transmit ASFV to domestic swine, *Sus scrofa domesticus* Erxleben (Artiodactyla: Suidae) populations with devastating consequences [9]. Infected swine shed ASFV directly and mortality rates approach 100% by acute hemorrhagic fever [10]. Meat and animal by-products from infected swine can provide sources of spread through global trade and human transport and a possible source of introduction to the western Hemisphere [11]. The USA is the world’s third-largest producer of pork products. In 2015, the USA produced more than 110 metric tons of pork products valued at $22 billion and supporting 550,000 USA jobs [12, 13]. Therefore, an introduction of ASFV to the USA could induce severe economic consequences and risk potential establishment in *O. turicata* populations. The global spread of ASFV poses the risk of its re-emergence in the Americas, and the potential transmission by *O*. *turicata* to domestic pigs (*Sus scrofa domesticus*) and feral swine (*Sus scrofa scrofa*).

The host range of *O. turicata* remains to be fully understood. Larvae, nymphs and adults of *O. turicata* acquire a blood meal rapidly as compared to hard ticks [14], and are rarely discovered feeding on hosts. The host list includes many diverse animals when considering the extensive fauna frequenting cavity environments where *O. turicata* has been collected [2]. Recent serological evidence of sampled feral swine indicated the animals were exposed to *B. turicatae*, providing indirect evidence of *O. turicata* blood-feeding on feral swine in Texas [15]. Additionally, photographic and field evidence confirmed feral swine visitations to *O. turicata* infested caves and rodent burrows. Parasitism of feral swine moving across the USA-Mexico transboundary region provides a potential pathway for the emergence of ASFV in the USA [16].

Argasid ticks, including *O. turicata*, may be found in peridomestic and domestic settings [1, 17], with their habitats overlapping that of feral swine. However, the extent of feral swine exposure to *O. turicata* in the USA is unknown. The omnivorous behavior, habitat association, and landscape usage of feral swine suggest there are opportunities for *O. turicata*-feral swine interactions [18–21]. Importantly, direct interaction between feral swine and domestic swine occurs [20, 22], which poses a concern for the spread of emerging pathogens in the USA. Therefore, it is important to develop approaches that will aid in surveillance efforts.

A knowledge gap exists in the antibody response generated in pigs after exposure to blood-feeding by *O. turicata*. Defining the humoral response against tick salivary proteins will facilitate surveillance studies to estimate exposure of feral swine to *O. turicata* feeding. In this study, we documented the feeding success of *O. turicata* on domestic pigs and evaluated antibody responses against tick salivary proteins. Animals were exposed to 100 ticks in each of the three challenges. IgG responses were assessed by enzyme-linked immunosorbent assay (ELISA) and immunoblotting to salivary gland extracts (SGE) from dissected ticks. Our findings indicated that domestic pigs seroconverted to *O. turicata* salivary proteins. These results suggest that a serological assay could be implemented for surveillance studies to determine the exposure of domestic pigs and feral swine to *O. turicata*.

## Methods

### *Ornithodoros turicata* colony

Adult and late-instar *O. turicata* nymphs used in this study originated from specimens collected in a natural cavern in Travis County, Texas, USA, in 1992 and are maintained in colony at the Tick Research Laboratory at Texas A&M AgriLife Research, College Station, TX, USA. Ticks were reared on young cockerels (*Gallus gallus*) as blood-meal host, and maintained under a 14:10 h (light:dark) photoperiod, temperature of 25.0 ± 3.0 °C, and relative humidity of 80–85%. Prior to feeding cohorts of *O. turicata* on pigs, they were starved for seven months.

### Host preparation

Four weaned domestic pigs (*S. s. domesticus*) weighing 15–20 kg were acquired from a commercial swine producer. The pigs were maintained at the Texas A&M University Veterinary Medical Research Park for the duration of the study in accordance with IACUC-approved AUP No. 2015-0089. This facility was specifically designed to maintain an ectoparasite free environment other than the ticks used in the study. Pigs were quarantined for two weeks during which they received standard veterinary preventative treatment in accordance with operating procedures of the research facility. Pigs underwent two positive reinforcement training sessions for acclimation to a sling apparatus. This method facilitated tick feeding and venous blood collection from the pigs.

### Tick challenge and blood collection

**Fig. 2:**
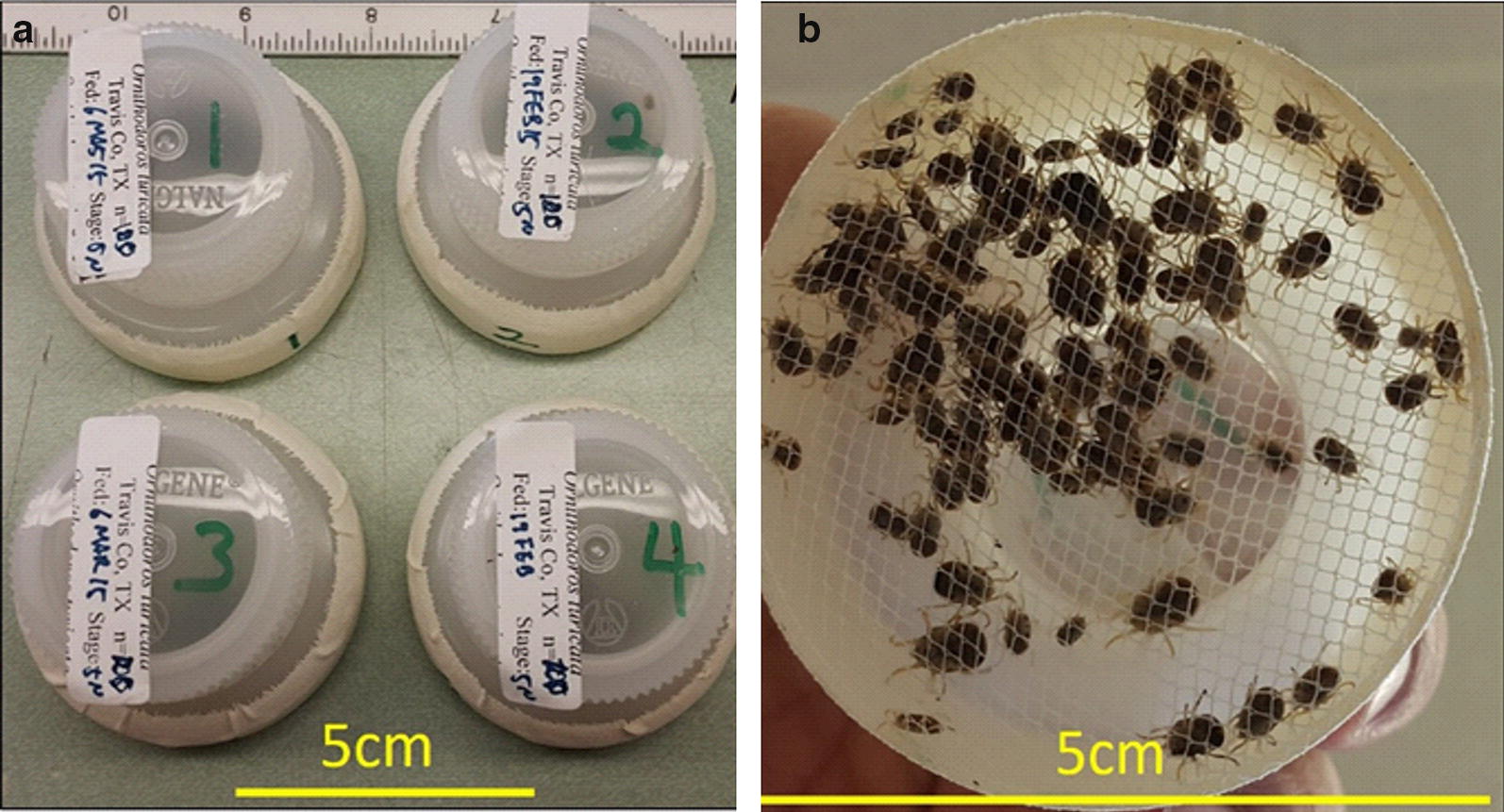
Feeding chambers used for *Ornithodoros turicata* challenges. One hundred *O. turicata* were fed on each pig. Ticks were placed in one of four feeding chambers (**a**) then randomly assigned to each pig subject. The feeding chamber was sealed with fabric (mesh size 2 mm) (**b**), through which the ticks could feed. Scale-bars are shown at the bottom of each image

Prior to feeding *O. turicata* on pigs, a pre-challenge serum sample was obtained from each animal. Ticks were fed three times on pigs in 2-week intervals (Table 1). Prior to each tick feeding, a blood sample was obtained from each animal. After the third tick feeding, a blood sample was collected from each animal weekly for four weeks. The final two blood samples were collected 28 days apart (Table 1). For each tick feeding, 100 unfed ticks that had completed four molts (adult and/or late-instar nymphs) were placed in a feeding chamber then randomly assigned to a pig subject. Feeding-chamber design was adapted from previous experiments where the chambers were constructed by removing the bottom of a 250 ml wide-mouth Nalgene bottle (Thermo Fisher Scientific, Waltham, MA, USA) to 4.5 cm from the top and sealing it with fabric having a mesh size of 2 mm through which the ticks could feed [23, 24] (Fig. 2). Feeding chambers were secured onto the backs of the pig subjects for 60 min using 3M VetRap (3M, Oakdale, MN, USA).

**Table 1:** Summary of the experimental timeline and procedures

Experimental day	Procedure
01	Pre-challenge blood sample collection
08	Tick challenge #1
21	Post-challenge blood sample #1
22	Tick challenge #2
35	Post-challenge blood sample #2
36	Tick challenge #3
49	Post-challenge blood sample #3
56	Post-challenge blood sample #4
63	Post-challenge blood sample #5
70	Post-challenge blood sample #6
98	Post-challenge blood sample #7
126	Post-challenge blood sample #8

Ten ml blood samples from each pig were collected over the course of the study (Table 1). The pre-challenge blood sample collected served as the control (baseline). The collected blood samples were allowed to clot at room temperature and centrifuged using a Variseal Model Vs6c centrifuge (Vulcan Tech, New York, NY, USA) at a 600×*g* for 10 min. The isolated serum samples were stored at − 20 °C until used for analyses.

### Preparation of tick SGE

We used SGE as the antigen to assess pig serological responses. Dissected ticks were flat (unfed) late stage nymphs and were reared similar to ticks used for parasitizing pigs. Also, SGE were generated using the modified methods derived from Canals et al. [25]. Each *O. turicata* specimen was placed into phosphate-buffered saline (PBS) with 5 mM magnesium chloride (MgCl_2_) and dissected using an Axio Stemi microscope (Zeiss, Munich, Germany). Salivary glands were removed using forceps and washed with PBS and 5 mM of MgCl_2_. Salivary glands were placed in 1.5 ml tube containing 100 µl of PBS and 5 mM of MgCl_2_, homogenized using a polypropylene pestle (Bel-Art Products, Wayne, NJ, USA), and centrifuged at 10,000×*g* for 5 min. Protein concentration of SGE supernatants were determined using Epoch Microplate Spectrophotometer and Gen5 Data Analysis software version 2.00.18 (BioTek, Winooski, VT, USA) with bovine serum albumin (Bio-Rad, Hercules, CA, USA) as a standard. SGE supernatants were stored at − 4 °C until needed.

### Enzyme-linked immunosorbent assay (ELISA)

ELISA using SGE was performed to evaluate serological responses over the duration of the study and to determine endpoint titers. Pre- and post-tick challenge serum samples were compared for each animal. For all assays, 96-well flat bottom Immulon 2 HB plates (Thermo Electron, Milford, MA, USA) were coated with 100 μl of coating buffer with 1 μg of SGE protein per well and incubated overnight at 4 °C. Plates were washed three times with PBS Tween-20 (1× PBS, 0.05% Tween-20). Each plate was blocked using ELISA diluent (PBS, 0.5% horse serum, 0.05% Tween-20, 0.001% dextran sulfate) at 100 μl per well and incubated for 1 h at room temperature. Plates were washed three times, 100 μl of pig sera from tick challenges were added to wells at a dilution of 1:100, and plates were incubated at room temperature for one hour. Each plate was washed three times as stated above, and 100 μl of the secondary antibody, anti-pig IgG Fc-HRP (Thermo Fisher Scientific, Waltham, MA, USA), was added to each well at 1:5000 dilution. After an hour of incubation at room temperature, each plate was washed three times, and 100 μl ELISA HRP substrate was added to each well and incubated at room temperature for 15 min. The optical density of each plate was evaluated at 405 nm absorbance using the Epoch Microplate Spectrophotometer and Gen5 Data Analysis software version 2.00.18 (BioTek, Winooski, VT, USA).

To determine endpoint titers, ELISA was performed using serum samples at a 1:100 to 1:512,000 dilution. Pre-challenge serum samples diluted 1:100 were also used. All samples were tested in triplicate and means and standard deviations determined. Samples were considered statistically significant if their mean optical density was more than three times the standard deviation of the mean of the pre-tick challenge serum sample, as previously reported [26, 27].

### Sodium dodecyl sulfate polyacrylamide gel electrophoresis (SDS-PAGE) and immunoblotting

Proteins were separated by SDS-PAGE and immunoblotting was performed to determine seroconversion, as described [28]. Three µg of SGE supernatant was electrophoresed on Mini-Protean TGX precast gels (Bio-Rad) at 80 volts for 90 min. Proteins were transferred to polyvinylidene fluoride (PVDF) membranes (Millipore, Billerica, MA, USA) using 100 volts for 60 min in a Mini Trans Blot system (Bio-Rad). PVDF membranes were blocked overnight with I-Block Protein-Based Blocking Reagent (Life Technologies, Grand Island, NY, USA). Immunoblots were probed with pig serum samples as primary antibodies at a 1:200 dilution for one hour. Membranes were washed with I-Block for one hour and anti-pig IgG-HRP (Life Technologies) was used as the secondary antibody at a 1:4000 dilution. Serological reactivity was determined with the Amersham Enhanced Chemiluminescence (ECL) Western Blotting System (GE Healthcare Bio-Science Corp., Piscataway, NJ, USA).

### Statistics

The statistical program JMP Pro 12 statistical software (SAS Co., Cary, NC, USA) was used. ANOVA and a Tukey’s honest significant difference (HSD) *post-hoc* test based on an alpha level of 0.05 were performed for all pairwise combinations to determine significant differences in total tick fed and post-blood-feeding weight increase between challenges, feeding chamber, and pig subjects.

## Results

### Evaluation of tick feeding

**Fig. 3:**
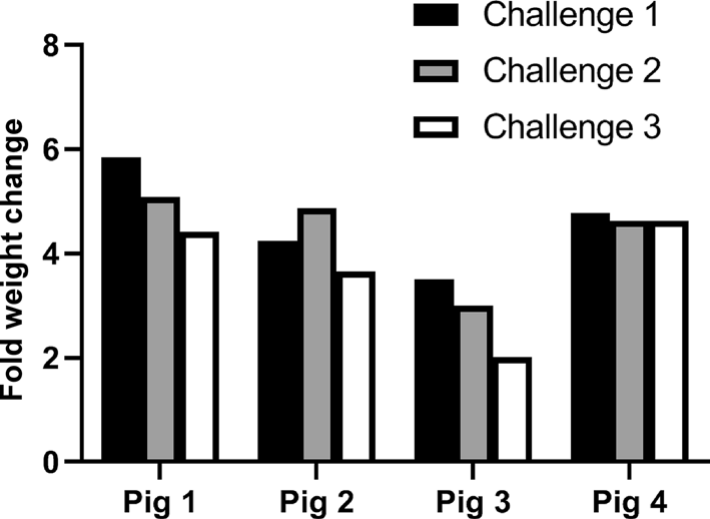
Fold weight change after feeding cohorts of 100 ticks on a given animal. Change in weight was determined by weighing pre- and post-fed tick cohorts. Shown are challenge 1 (black fill), 2 (grey fill) and 3 (white)

*Ornithodoros turicata* readily fed on pigs. For tick cohorts that fed on the four animals, we observed between a 2.01- to 5.85-fold change in weight after feeding (Fig. 3). The proportion of ticks that fed, and the weight increase per cohort were similar among the four pigs used in these experiments (*F*_(3, 8)_ = 1.556; *P* = 0.2739 for total ticks fed, and *F*_(3, 8)_ = 0.9568; *P* = 0.4584 for weight increase per cohort). Similarly, the fraction of ticks that fed, and the weight increase per cohort were not significantly different according to feeding chamber (*F*_(3, 8)_ = 1.8209; *P* = 0.2214 for total tick fed, and *F*_(3, 8)_ = 0.20901; *P* = 0.8873 for weight increase per cohort). The percent weight increase was not significantly different based on challenges (*F*_(2, 9)_ = 3.7277; *P* = 0.0662). However, the total number of ticks fed significantly differed between challenge dates (*F*_(2, 9)_ = 5.1758; *P* = 0.0319). Specifically, total ticks that fed during challenge 3 were significantly different from those of challenge 1 (Tukey’s HSD *P* = 0.0261). A proportional decrease in ticks fed of 14%, 14%, 16% and 7% for pigs 1 to 4, respectively, was noted between the first and third challenges. These findings suggest that repeated tick exposure to pigs may impact tick feeding success.

### Pig serological responses following *O. turicata* feeding

**Fig. 4:**
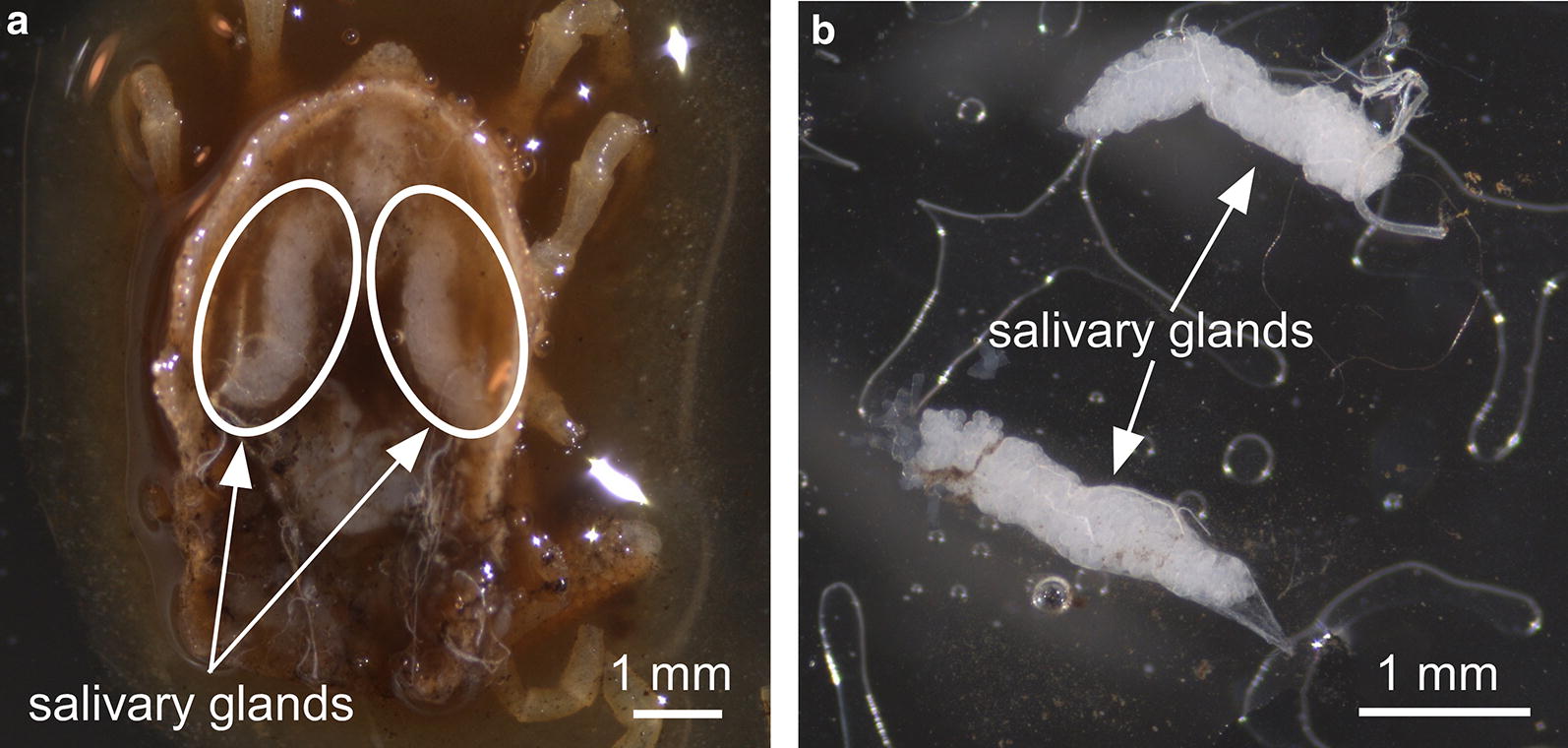
Dissection of *Ornithodoros turicata* salivary glands. Ticks were dissected, removing the midgut, which exposed the salivary glands (**a**). Salivary gland sets were excised and rinsed to remove residual midgut content (**b**). Scale-bars are shown in the bottom left of each image

**Fig. 5:**
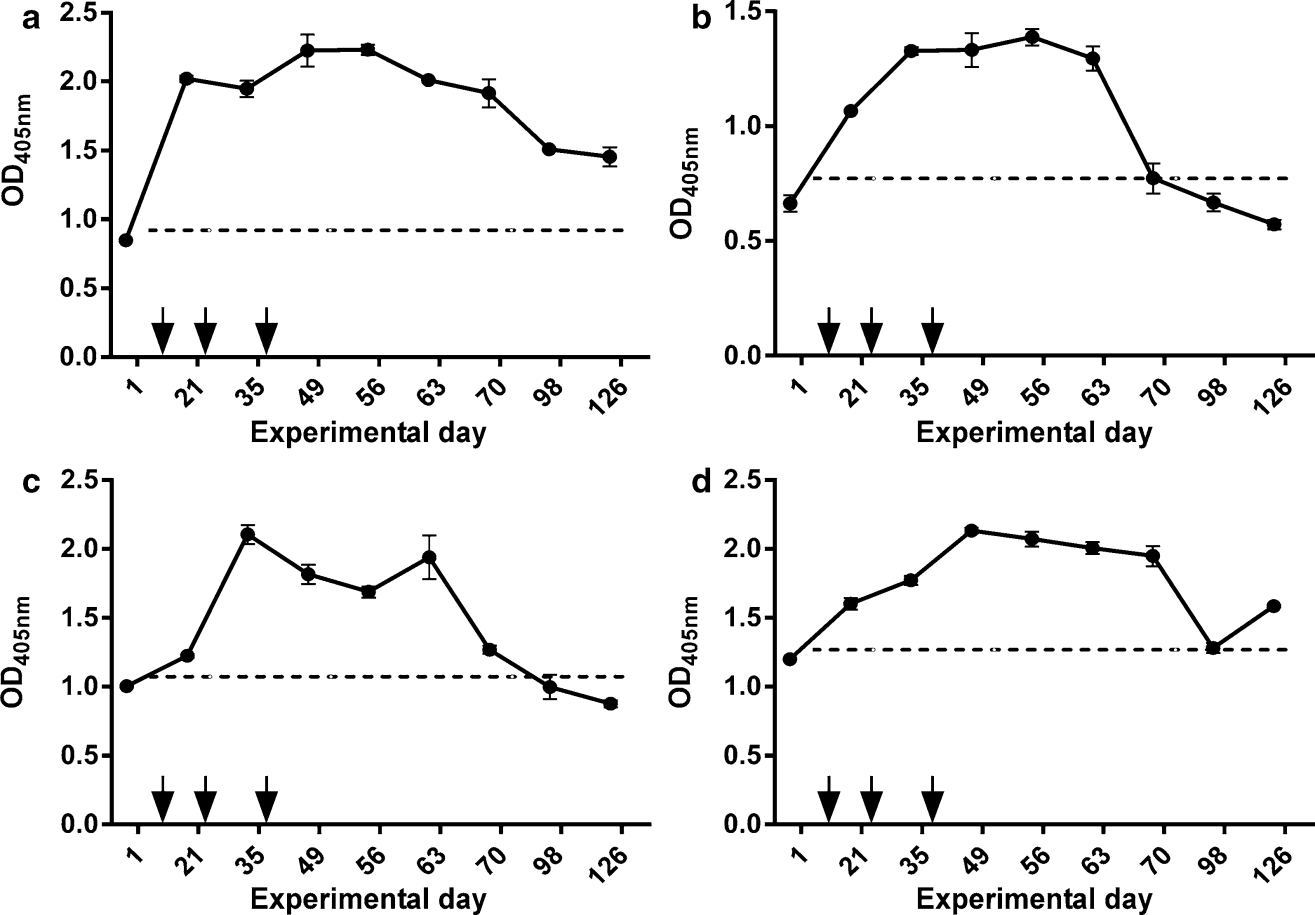
ELISA assessment of temporal serological responses to tick SGE. Serum samples from animals 1 (**a**), 2 (**b**), 3 (**c**) and 4 (**d**) were evaluated prior to (day 1) and for 90 days after the third tick challenge. Arrows denote the days of tick feedings. A sample was considered statistically significant (*P* ≤ 0.003) if their mean optical density was more than three times the standard deviation of the mean of the pre-tick challenge serum sample. This threshold is represented by a dotted line

To evaluate serological responses to tick salivary proteins, we dissected intact salivary glands from *O. turicata* (Fig. 4) and used them to generate SGE. Within 13 days after the first tick challenge (day 21 of the experimental design, Table 1), ELISA assays detected statistically significant (*P* ≤ 0.003) IgG responses from all pigs compared to the respective mean pre-challenge serum samples from each pig (Fig. 5a–d). Elevated IgG responses were detectable for a majority of the study (Fig. 5a–d). Pig 1 generated the most prolonged IgG response for the duration of the study (Fig. 5a) with statistically significant (*P* ≤ 0.003) detection occurring with the final serum sample tested. IgG responses of pig 2 were above the pre-challenge serum sample cut-off for 27 days after the third tick challenge, after which antibody responses were undetectable (Fig. 5b). Pig 3 generated a statistically significant (*P* ≤ 0.003) IgG response for 34 days after the third tick challenge (Fig. 5c). Pig 4 generated a prolonged IgG response that was above the pre-challenge cut-off until day 98 (Fig. 5d). At day 126, pig 4’s serological response rose above the pre-challenge cut-off. Endpoint titers were determined using post-challenge sera with the highest OD reading. Pig 2 generated the strongest response with a titer of 1:16,000, while pig 4 had the weakest response with a titer of 1:4000.

**Fig. 6:**
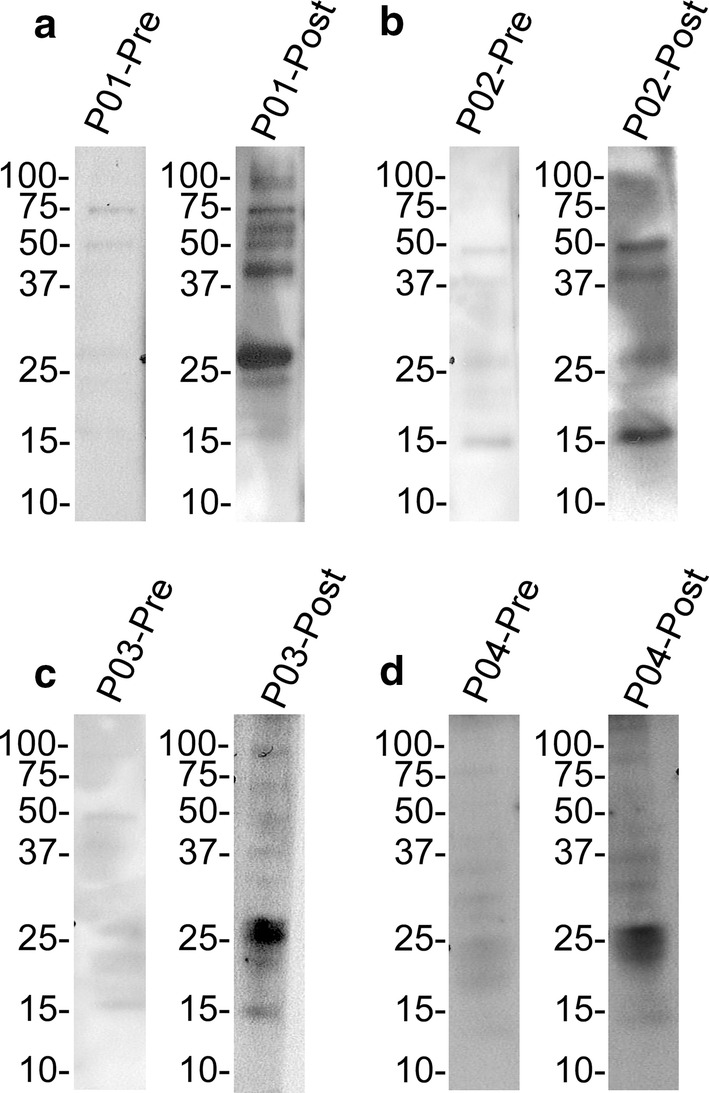
Immunoblot analysis of serum samples from pigs fed upon by *Ornithodoros turicata.* Pre- and post-exposure serum samples from animal 1 (**a**), 2 (**b**), 3 (**c**) and 4 (**d**) were used to evaluate seroconversion. Serological reactivity to a ~25 kDa protein was observed from the four animals. Molecular masses are indicated on the left of each immunoblot

The antigenic profile detected in pig serum samples was further assessed by immunoblotting using SGE from *O. turicata* (Fig. 6). Reactivity using serum samples prior to tick challenge was compared to serum samples collected 13 days after the third tick feeding, indicating the antigenicity of salivary secretions. Salivary antigens with molecular masses of ~ 15 to ~ 100 kDa were identified. All the pigs showed a strong IgG response to salivary proteins with a molecular weight of ~ 25 kDa. These results indicated that *O. turicata* salivary secretions were antigenic in domestic pigs.

## Discussion

Our study reports serological responses of pigs to *O. turicata* salivary antigens after repeatedly exposing the animals to the ticks. The animals seroconverted to *O. turicata* salivary proteins that ranged from ~ 15 to 100 kDa and this was sustained between 27 to 90 days after the third tick challenge. In previous work, humoral response of rabbits was evaluated after *O*. *turicata* feeding [29]. Wozniak et al. [29] reported an increase of IgG production in rabbits that were challenged with several *Ornithodoros* species. They fed *O*. *turicata*, *O. talaje* and *O. coriaceus* on rabbits bi-weekly and evaluated IgG responses to SGE by ELISA and immunoblotting. Each feeding involved 150 ticks from each *Ornithodoros* species, and the rabbits were exposed four times. Salivary gland proteins recognized by the rabbit antibodies ranged from ~ 15 to ~ 130 kDa, and endpoint titers were ~ 1:5120. However, the longevity of IgG responses after the fourth tick challenge were not reported. Similar to our findings, rabbits also developed serological responses to a salivary protein of ~ 25 kDa [29], but it is unknown whether these proteins are the same.

When we evaluated serum samples of each pig, differences between the serological assays were observed. For example, while serological responses from each animal were similar when assessed by ELISA, immunoblotting indicated differing reactivity between animals. Specifically, when we tested serum samples collected 13 days after the third challenge, pig 1 reacted with more antigens from SGE compared to the other animals. While not determined in our study, the haplotypic diversity of pig 1 may have affected the antigen repertoire recognized by this animal.

After the tick third challenge, we observed a weight loss between *O. turicata* ticks that fed compared to cohorts from the first two feedings due to fewer ticks feeding. A decline in successful tick feeding on repeatedly exposed animals is an indicator of acquired immunity against arthropods [30–33]. Riek et al. [34] reported hypersensitivity to *Rhipicephalus* (*Boophilus*) *microplus* salivary secretion elicited by heavy tick infestation, which resulted in histological changes of the skin at the site of attachment of two species of cattle, *Bos taurus* L. (Artiodactyla: Bovidae) and *Bos indicus* L. Similarly, Szabó & Bechara [35] reported that repeat exposures to *Rhipicephalus sanguineus* (*s.l.*) Latreille (Ixodida: Ixodidae) elicited strong skin inflammatory responses from both dogs and guinea pigs. These studies evaluated successful feeding of ixodid ticks, which lay cement, attach for days, and regurgitate midgut content. Moreover, ixodid salivary and midgut proteins are antigenic and have been targeted to interrupt tick feeding [36]. In comparison, *O. turicata* engorges within minutes of attachment, and it remains unknown if midgut content is regurgitated and an immune response induced, which could impact feeding success. Moreover, while salivary secretions are antigenic in *Ornithodoros* species, it is unclear as to whether the immune response generated against the proteins would impact subsequent tick feedings.

Numerous species of ixodid ticks feed on feral swine in Texas [15], however less is known about interactions between argasids and feral swine because they are rapid feeders that are rarely found attached to the host. Feral swine populations have become a serious threat throughout the southern USA, overlapping the known Florida and southwestern distributions of *O. turicata* [37]. Our studies suggest that feral swine could potentially serve as sentinels to detect the presence of *O. turicata* through serologic surveillance. This has been demonstrated in previous work demonstrating the antigenicity of salivary secretions from *O. erraticus* and *O. moubata* in swine [25, 38]. This approach was also used to measure tick exposure in swine populations in the Iberian Peninsula, Sardinia, and Madagascar as part of assessing ASFV epidemiology and progress toward disease control and elimination [39–42]. Consequently, to improve surveillance efforts, it is important to develop an accurate molecular assay to differentiate exposure of feral swine to *Ornithodoros* species and ixodid ticks. With the availability of the *O. turicata* salivary gland transcriptome [43], future proteomic work will identify the tick salivary antigens recognized by the vertebrate immune response. This will be an important first step toward the development of a specific assay to evaluate the exposure of domestic and wild pigs to *O. turicata* salivary proteins.

## Conclusions

This study describes a successful animal model to study molecular interactions between *Ornithodoros* ticks and pigs that could aid in further development of methods to study soft tick ecology, as well as in establishing early detection and prevention of ASF entry into the USA through wildlife.

## Data Availability

The datasets generated in this study are available upon request.

## Abbreviations

ASF: African swine fever
ASFV: African swine fever virus
ELISA: enzyme-linked immunosorbent assay
SGE: salivary gland extract
IACUC: Institutional Animal Care and Use Committee
AUP: animal use protocol
PBS: phosphate-buffered saline
HRP: horseradish peroxidase
ANOVA: analysis of variance
HSD: honest significant difference
